# Synthesis and crystal structure of (2*E*)-1-[3,5-bis­(benz­yloxy)phen­yl]-3-(4-eth­oxy­phen­yl)prop-2-en-1-one

**DOI:** 10.1107/S2056989024007552

**Published:** 2024-08-06

**Authors:** K. R. Jeyashri, G. Logeshwari, U. Rajapandiyan, K. Sivakumar, S. Selvanayagam, H. Manikandan, K. Kaviyarasu

**Affiliations:** a Department of Chemistry Annamalai University, Annamalainagar Chidambaram 608 002 India; b Department of Chemistry Sri Chandrasekharendra Saraswathi Viswa Mahavidyalaya, (Deeded to be University) Kanchipuram 631 561 India; c PG & Research Department of Physics, Government Arts College, Melur 625 106, India; d Nanosciences/Nanotechnology Laboratories University of South Africa (UNISA) Pretoria South Africa; University of Aberdeen, United Kingdom

**Keywords:** crystal structure, chalcone derivative, C—H⋯O inter­actions, Hirshfeld surface analysis

## Abstract

In the title compound, C_31_H_28_O_4_, the phenyl rings of the chalcone unit subtend a dihedral angle of 26.43 (10)°. The phenyl rings of the pendant benz­yloxy groups are orientated at 75.57 (13) and 75.70 (10)° with respect to their attached ring. In the crystal, weak C—H⋯O and C—H⋯π inter­actions link the mol­ecules. The inter­molecular inter­actions were qu­anti­fied and analysed using Hirshfeld surface analysis, which showed a breakdown into H⋯H (49.8%), H⋯C/C⋯H (33.8%) and H⋯O/O⋯H (13.6%) inter­actions with other types making negligible contributions.

## Abstract 2

In the title compound, the phenyl rings of the chalcone unit subtend a dihedral angle of 26.43 (10)°. The phenyl rings of the pendant benz­yloxy groups are orientated at 75.57 (13) and 75.70 (10)° with respect to their attached ring. In the crystal, weak C—H⋯O and C—H⋯π inter­actions link the mol­ecules, forming *C*(15) chains propagating along [101].

## 1. Chemical context

Chalcones incorporate an α,β-unsaturated carbonyl (enone) bridge connecting two aromatic rings. The chalcone scaffold exhibits anti-cancer efficacy on various human cancer cells (Zhuang *et al.*, 2017 ▸; Liu *et al.*, 2022 ▸). DrugBank lists three chalcone-based drugs namely hesperidin methyl­chalcone (DrugBank: DB15943), di­hydroxy­meth­oxy­chalcone (DB14122) and 3-(4-hy­droxy­phen­yl)prop-2-en-1-one (DB07500). In general, the anti­cancer efficacy of chalcones is enhanced by attaching different substitutents at ring *A* of the chalcone, which is attached to the C=O group (Mai *et al.*, 2014 ▸). As part of our studies in this area, we have prepared and undertaken a single-crystal X-ray diffraction study of the title compound, C_31_H_28_O_4_, (I), and the results are presented here.
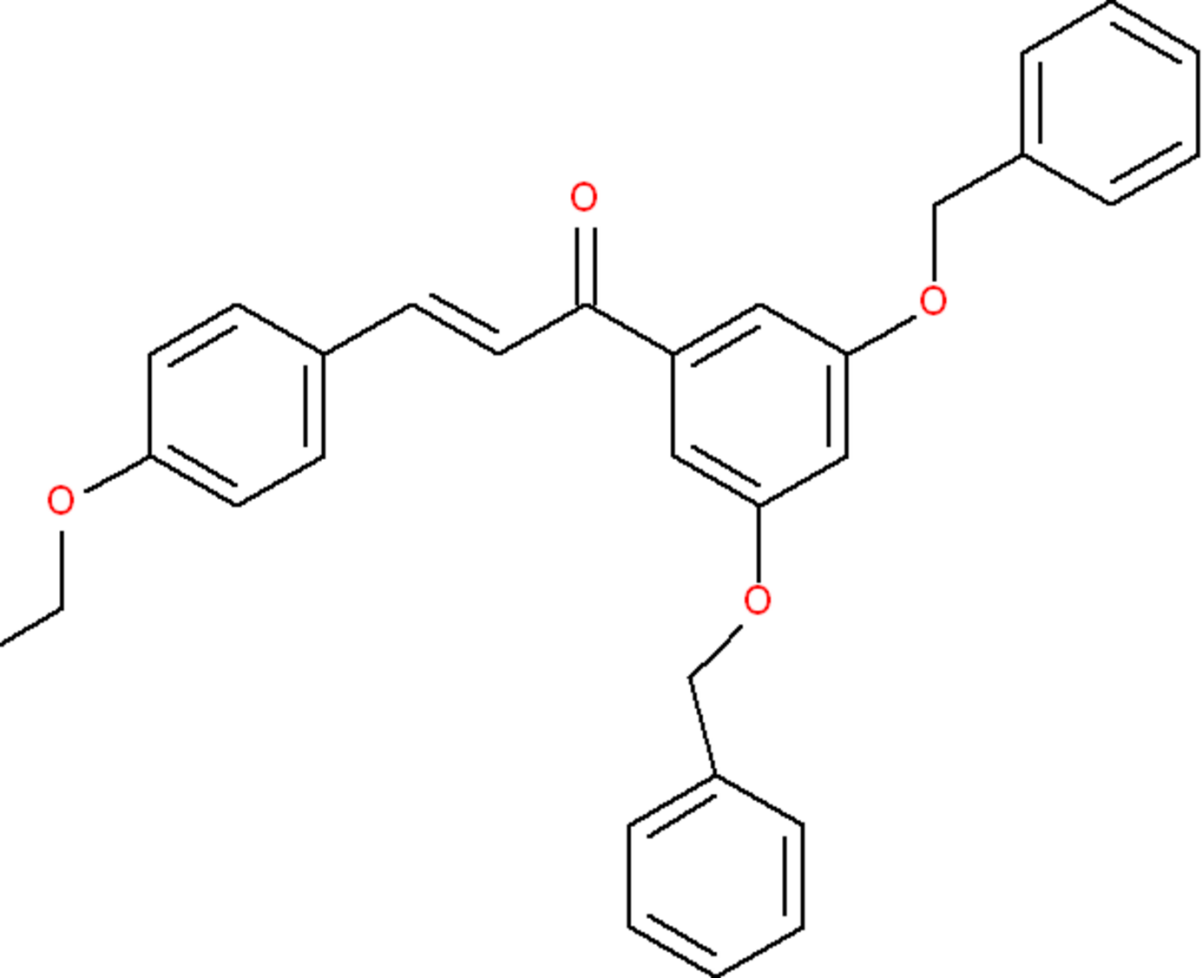


## 2. Structural commentary

**Figure 1:**
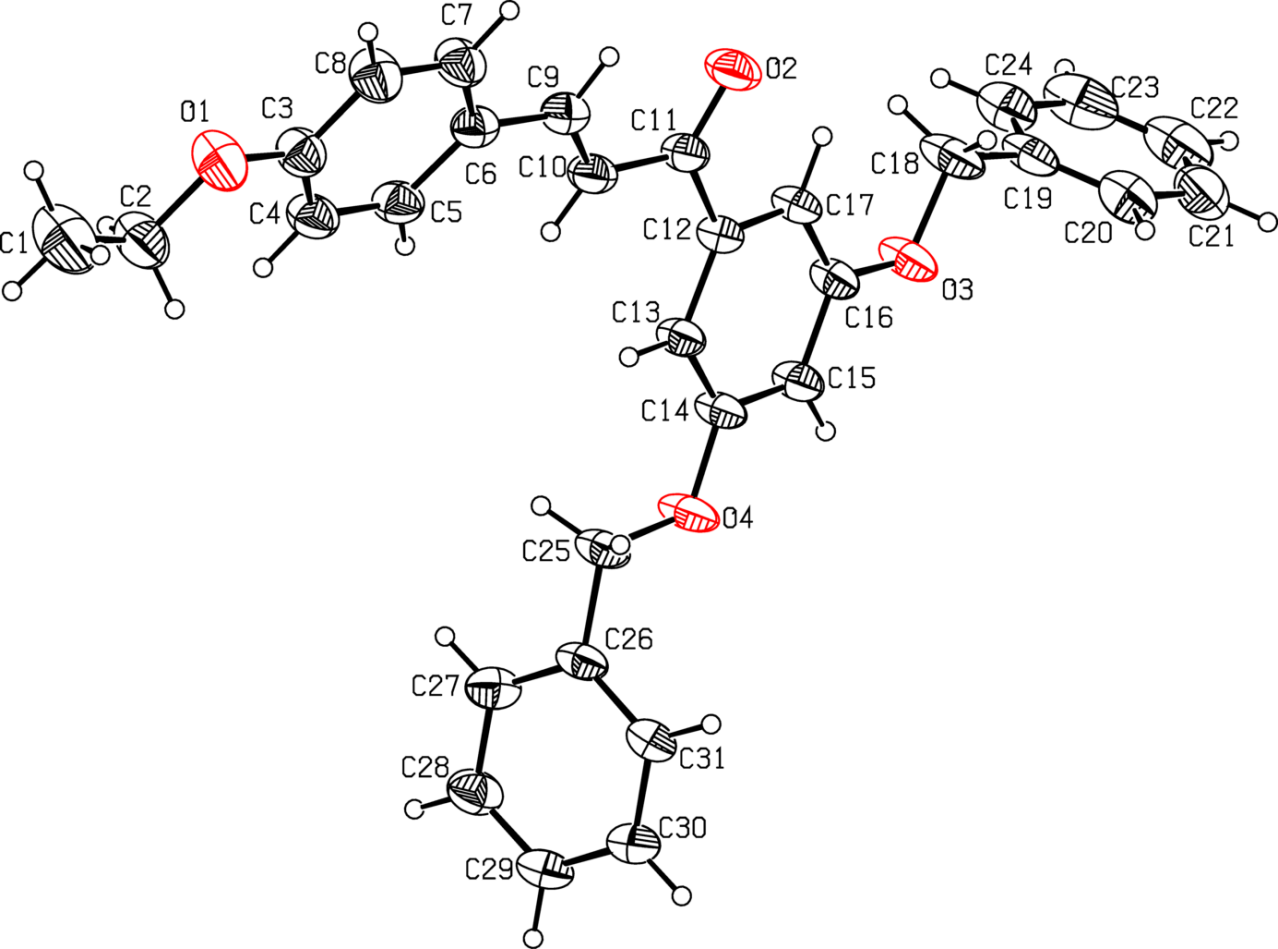
The mol­ecular structure of (I) with displacement ellipsoids drawn at the 30% probability level.

The mol­ecular structure of (I) is illustrated in Fig. 1 ▸. The C12–C17 and C19–C24 phenyl rings of the chalcone unit subtend a dihedral angle of 26.43 (10)°: the most significant twist occurs about the C11—C12 bond, as indicated by the C10—C11—C12—C13 torsion angle of −13.0 (3)°. The dihedral angles between the C19–C24 and C26–C31 pendant phenyl rings and their attached C12–C17 ring are 75.57 (13) and 75.70 (10)°, respectively. The C3—O1—C2—C1, C14—O4—C25—C26 and C16—O3—C18—C19 torsion angles of 177.2 (3), 176.21 (18) and 179.5 (2)°, respectively, indicate an *anti* conformation in each case.

## 3. Supra­molecular features

**Figure 2:**
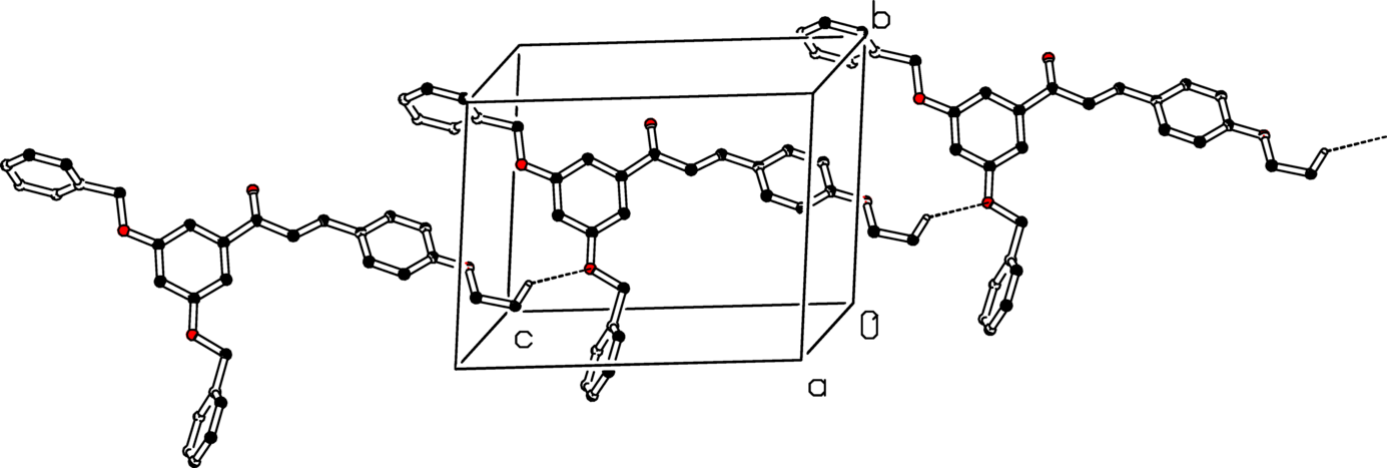
Detail of the packing of (I) showing C—H⋯O inter­actions as dashed lines. For clarity H atoms not involved in these hydrogen bonds have been omitted.

**Figure 3:**
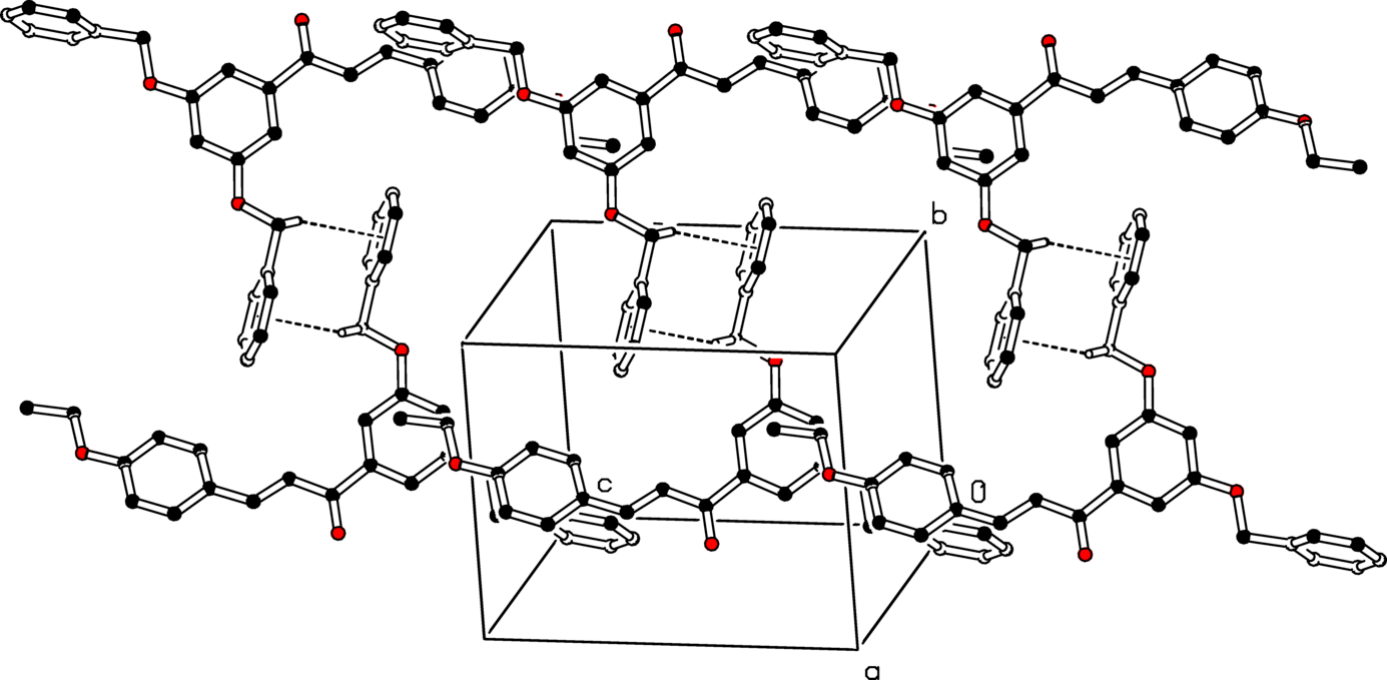
Detail of the packing of (I) showing C—H⋯π inter­actions as dashed lines.

In the crystal of (I), the mol­ecules associate *via* weak C—H⋯O inter­actions (Table 1 ▸), forming *C*(15) chains propagating along [101] (Fig. 2 ▸). In addition, inversion-related mol­ecules are linked by pairwise weak C—H⋯π inter­actions (Fig. 3 ▸).

## 4. Hirshfeld surface analysis

**Figure 4:**
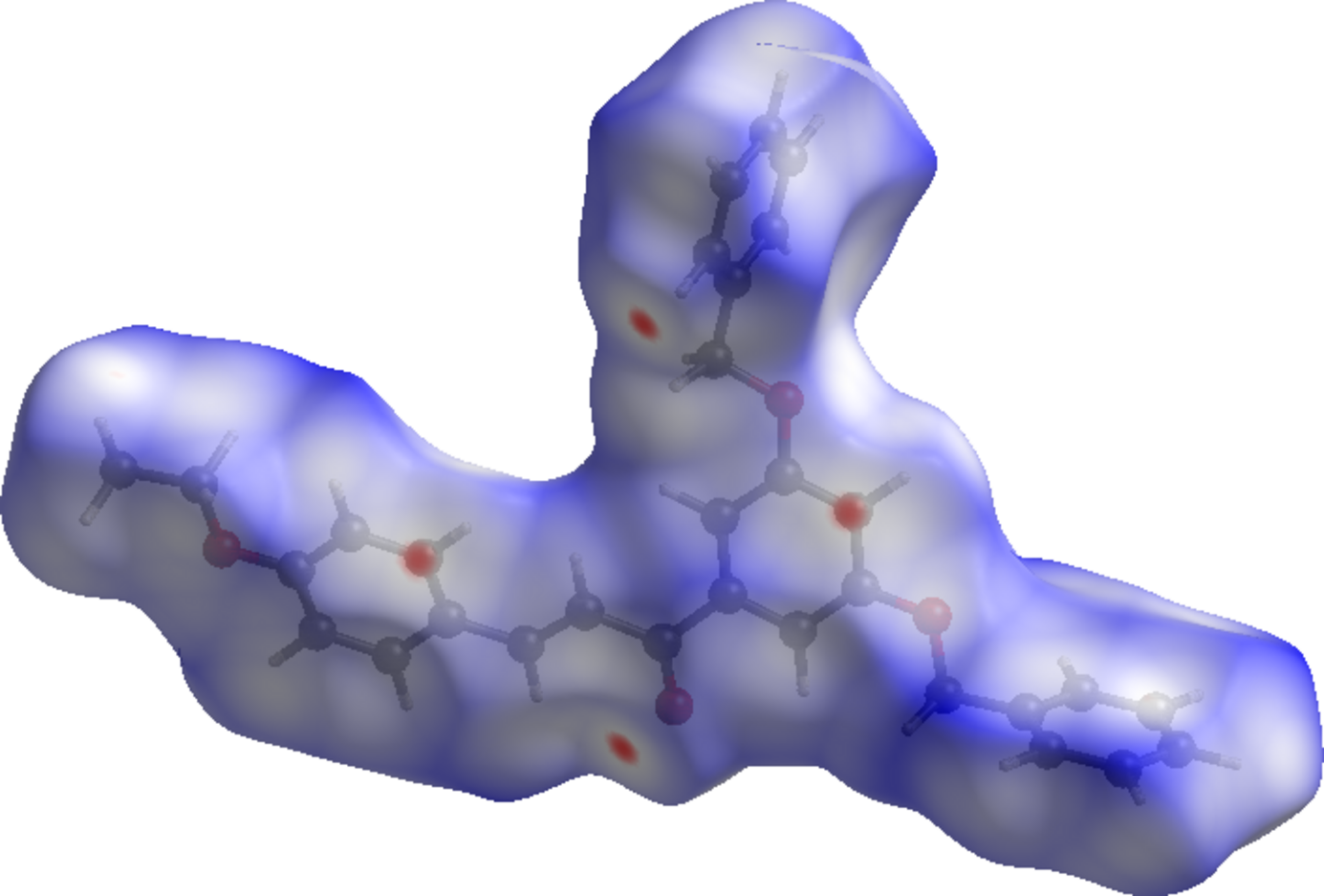
A view of the Hirshfeld surface mapped over *d*_norm_ for (I).

To further characterize the inter­molecular inter­actions in (I), a Hirshfeld surface analysis was performed using *Crystal Explorer 21* (Spackman *et al.*, 2021 ▸) and the associated two dimensional fingerprint plots were generated. The HS mapped over *d*_norm_ in the range −0.09 to +1.53 a.u. is illustrated in Fig. 4 ▸, using colours to indicate contacts that are shorter (red areas), equal to (white areas), or longer than (blue areas) the sum of the van der Waals radii (Ashfaq *et al.*, 2021 ▸).

**Figure 5:**
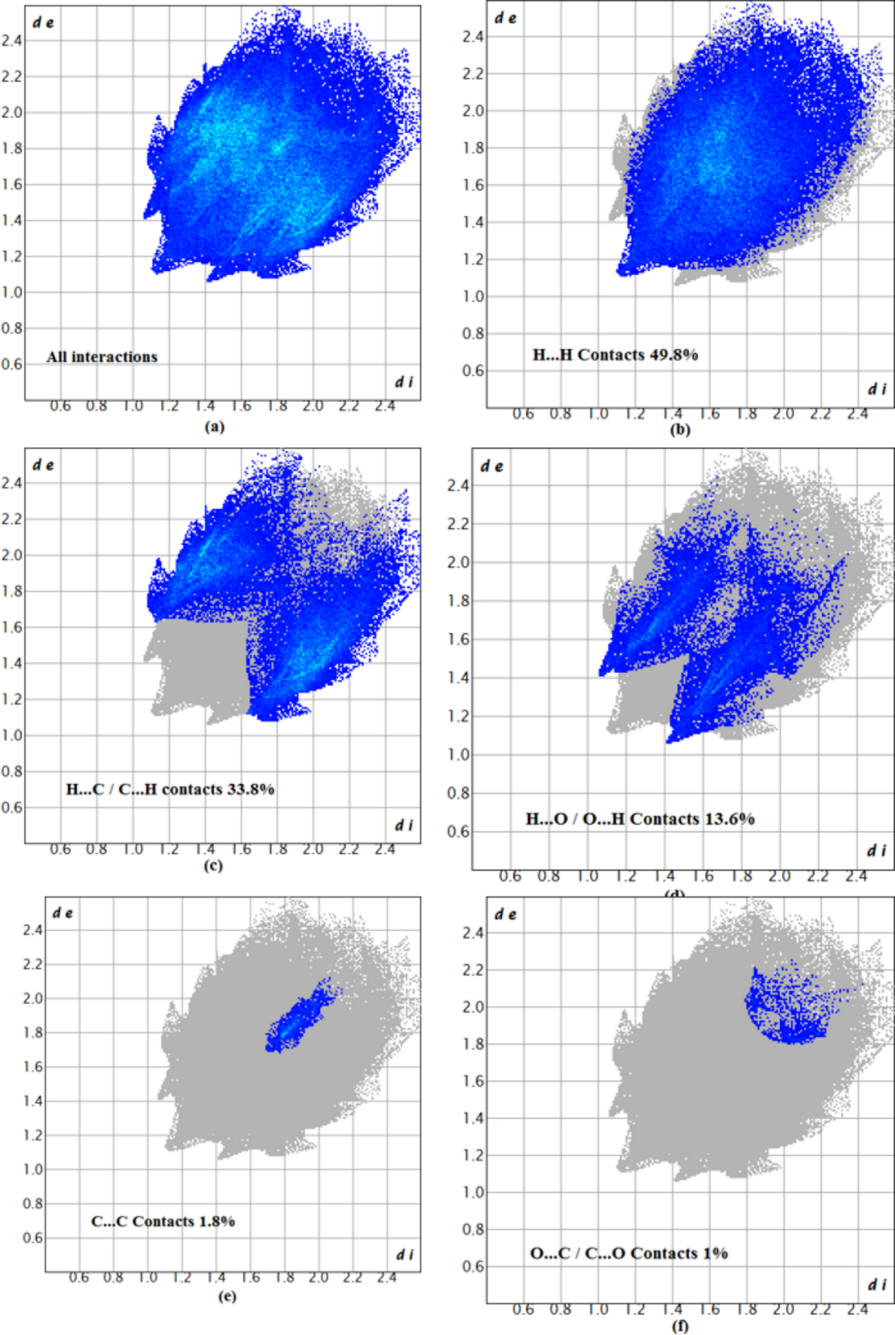
Two-dimensional fingerprint plots for (I), showing (*a*) all inter­actions, and delineated into (*b*) H⋯H, (*c*) H⋯C/C⋯H, (*d*) H⋯O/O⋯H, (*e*) C⋯C and (*f*) O⋯C/C⋯O inter­actions.

The overall two-dimensional fingerprint plot, Fig. 5 ▸*a*, and those delineated into H⋯H inter­actions (49.8%), H⋯C/C⋯H (33.8%), H⋯O/O⋯H (13.6%), C⋯C (1.8%) and O⋯C/C⋯O (1%) inter­actions are illustrated in Fig. 5 ▸*b*–*f*, respectively, together with their relative contributions to the HS. The most important inter­action is H⋯H, which is reflected in Fig. 5 ▸*b* as widely scattered points of high density due to the large hydrogen content of the mol­ecule with the tip at *d*_e_ = *d*_i_ = 1.10 Å. As a result of the presence of C—H⋯O inter­actions, the H⋯O/O⋯H contacts contribute 13.6% to the overall crystal packing, as reflected in Fig. 5 ▸*d* with the tips at *d*_e_ + *d*_i_ = 2.50 Å.

## 5. Synthesis and crystallization

Equimolar concentrations of 3,5-di­benzyl­oxyaceto­phenone and 4-eth­oxy­benzaldehyde were dissolved in ethanol in separate reaction flasks and then mixed. Drop by drop, utilizing a magnetic stirring device, 2 ml of 10% sodium hydroxide in water were introduced at room temperature. The course of the process was tracked using thin-layer chromatography. After the process was complete, the resulting product was placed on crushed ice. The finished product was vacuum-filtered, dried, and then recrystallized from ethanol solution to yield colourless blocks of the title compound.

IR (cm^−1^): 3032 aromatic C—H stretch, 2934 and 2875 aliphatic C—H stretch, 1651 C=O stretch, 1568 aromatic ring C=C stretch (see table in the supporting information).

## 6. Refinement

Crystal data, data collection and structure refinement details are summarized in Table 2 ▸. H atoms were placed in idealized positions and allowed to ride on their parent atoms: C—H = 0.93–0.97 Å, with *U*_iso_(H) = 1.5*U*_eq_(C-meth­yl) and 1.2*U*_eq_(C) for other H atoms.

## Supplementary Material

Crystal structure: contains datablock(s) I, global. DOI: 10.1107/S2056989024007552/hb8103sup1.cif

Structure factors: contains datablock(s) I. DOI: 10.1107/S2056989024007552/hb8103Isup2.hkl

Supporting information file. DOI: 10.1107/S2056989024007552/hb8103Isup3.cml

CCDC reference: 2182630

Additional supporting information:  crystallographic information; 3D view; checkCIF report

## Figures and Tables

**Table 1:** Hydrogen-bond geometry (Å, °) *Cg*1 is the centroid of the C26–C31 ring.

*D*—H⋯*A*	*D*—H	H⋯*A*	*D*⋯*A*	*D*—H⋯*A*
C1—H1*A*⋯O4^i^	0.96	2.64	3.227 (3)	119
C25—H25*B*⋯*Cg*1^ii^	0.97	2.68	3.398 (2)	132

Symmetry codes: (i) 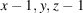; (ii) 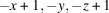.

**Table 2:** Experimental details

Crystal data	
Chemical formula	C_31_H_28_O_4_
*M*_r_	464.53
Crystal system, space group	Triclinic, *P* 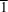
Temperature (K)	298
*a*, *b*, *c* (Å)	9.0494 (9), 10.0326 (11), 14.6932 (15)
α, β, γ (°)	100.153 (3), 107.292 (3), 90.789 (4)
*V* (Å^3^)	1250.6 (2)
*Z*	2
Radiation type	Mo *K*α
μ (mm^−1^)	0.08
Crystal size (mm)	0.31 × 0.23 × 0.19

Data collection	
Diffractometer	Bruker D8 Quest XRD
Absorption correction	–
No. of measured, independent and observed [*I* > 2σ(*I*)] reflections	30243, 5428, 3607
*R*_int_	0.037
(sin θ/λ)_max_ (Å^−1^)	0.638

Refinement	
*R*[*F*^2^ > 2σ(*F*^2^)], *wR*(*F*^2^), *S*	0.057, 0.178, 1.05
No. of reflections	5428
No. of parameters	317
H-atom treatment	H-atom parameters constrained
Δρ_max_, Δρ_min_ (e Å^−3^)	0.36, −0.18

Computer programs: *APEX3* and *SAINT* (Bruker, 2017 ▸), *SHELXT2018/2* (Sheldrick, 2015*a* ▸), *SHELXL2019/3* (Sheldrick, 2015*b* ▸), *ORTEP-3 for Windows* (Farrugia, 2012 ▸) and *PLATON* (Spek, 2020 ▸).

## supplementary crystallographic information

### (2E)-1-[3,5-Bis(benzyloxy)phenyl]-3-(4-ethoxyphenyl)prop-2-en-1-one Crystal data

**Table:**

C_31_H_28_O_4_	*Z* = 2
*M**_r_* = 464.53	*F*(000) = 492
Triclinic, *P*1	*D*_x_ = 1.234 Mg m^−^^3^
*a* = 9.0494 (9) Å	Mo *K*α radiation, λ = 0.71073 Å
*b* = 10.0326 (11) Å	Cell parameters from 9430 reflections
*c* = 14.6932 (15) Å	θ = 2.3–26.6°
α = 100.153 (3)°	µ = 0.08 mm^−^^1^
β = 107.292 (3)°	*T* = 298 K
γ = 90.789 (4)°	Block, colourless
*V* = 1250.6 (2) Å^3^	0.31 × 0.23 × 0.19 mm

### (2E)-1-[3,5-Bis(benzyloxy)phenyl]-3-(4-ethoxyphenyl)prop-2-en-1-one Data collection

**Table:**

Bruker D8 Quest XRD diffractometer	*R*_int_ = 0.037
Detector resolution: 7.3910 pixels mm^-1^	θ_max_ = 27.0°, θ_min_ = 2.3°
ω and Phi Scans scans	*h* = −11→11
30243 measured reflections	*k* = −12→12
5428 independent reflections	*l* = −18→18
3607 reflections with *I* > 2σ(*I*)	

### (2E)-1-[3,5-Bis(benzyloxy)phenyl]-3-(4-ethoxyphenyl)prop-2-en-1-one Refinement

**Table:**

Refinement on *F*^2^	0 restraints
Least-squares matrix: full	Hydrogen site location: inferred from neighbouring sites
*R*[*F*^2^ > 2σ(*F*^2^)] = 0.057	H-atom parameters constrained
*wR*(*F*^2^) = 0.178	*w* = 1/[σ^2^(*F*_o_^2^) + (0.0755*P*)^2^ + 0.344*P*] where *P* = (*F*_o_^2^ + 2*F*_c_^2^)/3
*S* = 1.05	(Δ/σ)_max_ < 0.001
5428 reflections	Δρ_max_ = 0.36 e Å^−^^3^
317 parameters	Δρ_min_ = −0.18 e Å^−^^3^

### (2E)-1-[3,5-Bis(benzyloxy)phenyl]-3-(4-ethoxyphenyl)prop-2-en-1-one Special details

**Table:**

Geometry. All esds (except the esd in the dihedral angle between two l.s. planes) are estimated using the full covariance matrix. The cell esds are taken into account individually in the estimation of esds in distances, angles and torsion angles; correlations between esds in cell parameters are only used when they are defined by crystal symmetry. An approximate (isotropic) treatment of cell esds is used for estimating esds involving l.s. planes.

### (2E)-1-[3,5-Bis(benzyloxy)phenyl]-3-(4-ethoxyphenyl)prop-2-en-1-one Fractional atomic coordinates and isotropic or equivalent isotropic displacement parameters (Å^2^)

**Table:**

	*x*	*y*	*z*	*U*_iso_*/*U*_eq_	
O1	−0.14058 (19)	0.34912 (19)	−0.00351 (12)	0.0891 (5)	
O2	0.16186 (19)	0.72402 (15)	0.58657 (11)	0.0798 (5)	
O3	0.57910 (18)	0.67306 (14)	0.89498 (11)	0.0758 (5)	
O4	0.58288 (19)	0.27603 (13)	0.68226 (10)	0.0781 (5)	
C1	−0.1307 (4)	0.2055 (4)	−0.1454 (2)	0.1262 (13)	
H1A	−0.151371	0.280342	−0.178933	0.189*	
H1B	−0.226967	0.158707	−0.150531	0.189*	
H1C	−0.069164	0.144122	−0.173877	0.189*	
C2	−0.0474 (3)	0.2556 (3)	−0.04420 (19)	0.0904 (8)	
H2A	−0.027085	0.180610	−0.009604	0.109*	
H2AB	0.051245	0.301090	−0.038289	0.109*	
C3	−0.0852 (2)	0.4030 (2)	0.09240 (16)	0.0674 (5)	
C4	0.0551 (3)	0.3730 (2)	0.15297 (17)	0.0692 (6)	
H4	0.117713	0.313490	0.128230	0.083*	
C5	0.1007 (2)	0.4316 (2)	0.24907 (16)	0.0653 (5)	
H5	0.194960	0.410658	0.288762	0.078*	
C6	0.0118 (2)	0.52131 (19)	0.29011 (15)	0.0581 (5)	
C7	−0.1280 (2)	0.5512 (2)	0.22718 (17)	0.0706 (6)	
H7	−0.189955	0.611871	0.251480	0.085*	
C8	−0.1756 (3)	0.4931 (3)	0.13069 (17)	0.0774 (6)	
H8	−0.269403	0.514352	0.090561	0.093*	
C9	0.0606 (2)	0.5819 (2)	0.39198 (15)	0.0611 (5)	
H9	−0.000981	0.647173	0.411977	0.073*	
C10	0.1840 (2)	0.5545 (2)	0.46044 (15)	0.0619 (5)	
H10	0.247017	0.488795	0.442843	0.074*	
C11	0.2253 (2)	0.62323 (18)	0.56229 (15)	0.0572 (5)	
C12	0.3502 (2)	0.57021 (17)	0.63744 (14)	0.0535 (4)	
C13	0.4072 (2)	0.44298 (18)	0.61769 (14)	0.0569 (5)	
H13	0.372300	0.389726	0.555824	0.068*	
C14	0.5168 (2)	0.39755 (18)	0.69199 (15)	0.0597 (5)	
C15	0.5686 (2)	0.47618 (19)	0.78386 (14)	0.0613 (5)	
H15	0.639925	0.443738	0.833530	0.074*	
C16	0.5144 (2)	0.60315 (19)	0.80212 (14)	0.0584 (5)	
C17	0.4042 (2)	0.65032 (18)	0.72948 (14)	0.0584 (5)	
H17	0.366639	0.735155	0.742260	0.070*	
C18	0.5246 (3)	0.8021 (2)	0.92022 (18)	0.0908 (8)	
H18A	0.413970	0.793221	0.911087	0.109*	
H18B	0.542890	0.862592	0.879393	0.109*	
C19	0.6105 (3)	0.8580 (2)	1.02462 (17)	0.0718 (6)	
C20	0.5696 (3)	0.8133 (3)	1.0964 (2)	0.0881 (7)	
H20	0.490407	0.745243	1.080563	0.106*	
C21	0.6443 (4)	0.8678 (3)	1.1924 (2)	0.1037 (10)	
H21	0.615398	0.836038	1.240799	0.124*	
C22	0.7596 (4)	0.9672 (4)	1.2169 (2)	0.1080 (11)	
H22	0.808981	1.004115	1.281899	0.130*	
C23	0.8029 (3)	1.0130 (3)	1.1459 (3)	0.1034 (10)	
H23	0.882143	1.081096	1.162282	0.124*	
C24	0.7284 (3)	0.9579 (3)	1.0495 (2)	0.0852 (7)	
H24	0.758359	0.988800	1.001161	0.102*	
C25	0.5493 (3)	0.19191 (19)	0.58876 (15)	0.0664 (6)	
H25A	0.574903	0.241425	0.543764	0.080*	
H25B	0.439988	0.162010	0.563792	0.080*	
C26	0.6465 (2)	0.07200 (18)	0.60074 (14)	0.0586 (5)	
C27	0.7704 (3)	0.0568 (2)	0.56452 (17)	0.0686 (6)	
H27	0.794339	0.121305	0.531744	0.082*	
C28	0.8599 (3)	−0.0535 (2)	0.57641 (18)	0.0731 (6)	
H28	0.942723	−0.062797	0.551033	0.088*	
C29	0.8279 (3)	−0.1484 (2)	0.62481 (17)	0.0698 (6)	
H29	0.889257	−0.221547	0.633478	0.084*	
C30	0.7042 (3)	−0.1351 (2)	0.66067 (18)	0.0749 (6)	
H30	0.681177	−0.199693	0.693631	0.090*	
C31	0.6136 (3)	−0.0260 (2)	0.64803 (17)	0.0705 (6)	
H31	0.529072	−0.018708	0.671861	0.085*	

### (2E)-1-[3,5-Bis(benzyloxy)phenyl]-3-(4-ethoxyphenyl)prop-2-en-1-one Atomic displacement parameters (Å^2^)

**Table:**

	*U*^11^	*U*^22^	*U*^33^	*U*^12^	*U*^13^	*U*^23^
O1	0.0745 (10)	0.1103 (13)	0.0696 (10)	0.0256 (9)	0.0120 (8)	−0.0008 (9)
O2	0.0974 (11)	0.0644 (9)	0.0736 (10)	0.0421 (8)	0.0194 (8)	0.0118 (7)
O3	0.0805 (10)	0.0589 (8)	0.0680 (9)	0.0309 (7)	0.0050 (7)	−0.0116 (7)
O4	0.0979 (11)	0.0489 (8)	0.0667 (9)	0.0363 (7)	0.0013 (8)	−0.0048 (6)
C1	0.113 (2)	0.159 (3)	0.085 (2)	0.057 (2)	0.0089 (18)	−0.001 (2)
C2	0.0876 (17)	0.0991 (19)	0.0795 (17)	0.0304 (15)	0.0240 (14)	0.0043 (14)
C3	0.0615 (12)	0.0707 (13)	0.0659 (13)	0.0100 (10)	0.0160 (10)	0.0074 (10)
C4	0.0655 (13)	0.0660 (13)	0.0748 (14)	0.0222 (10)	0.0188 (11)	0.0128 (11)
C5	0.0616 (12)	0.0615 (12)	0.0712 (14)	0.0188 (9)	0.0138 (10)	0.0181 (10)
C6	0.0555 (11)	0.0540 (10)	0.0658 (12)	0.0094 (9)	0.0167 (9)	0.0164 (9)
C7	0.0589 (12)	0.0798 (14)	0.0729 (14)	0.0245 (11)	0.0189 (11)	0.0143 (11)
C8	0.0568 (12)	0.0998 (17)	0.0706 (14)	0.0260 (12)	0.0119 (11)	0.0151 (12)
C9	0.0633 (12)	0.0542 (11)	0.0673 (13)	0.0133 (9)	0.0193 (10)	0.0154 (9)
C10	0.0686 (12)	0.0507 (10)	0.0665 (13)	0.0186 (9)	0.0186 (10)	0.0134 (9)
C11	0.0623 (11)	0.0447 (9)	0.0647 (12)	0.0136 (8)	0.0189 (9)	0.0105 (8)
C12	0.0567 (10)	0.0415 (9)	0.0623 (11)	0.0122 (8)	0.0182 (9)	0.0090 (8)
C13	0.0650 (11)	0.0412 (9)	0.0581 (11)	0.0133 (8)	0.0129 (9)	0.0019 (8)
C14	0.0653 (12)	0.0403 (9)	0.0672 (12)	0.0183 (8)	0.0143 (10)	0.0025 (8)
C15	0.0645 (12)	0.0494 (10)	0.0616 (12)	0.0196 (9)	0.0101 (9)	0.0029 (9)
C16	0.0613 (11)	0.0463 (10)	0.0603 (11)	0.0135 (8)	0.0139 (9)	−0.0022 (8)
C17	0.0619 (11)	0.0413 (9)	0.0684 (12)	0.0170 (8)	0.0186 (10)	0.0023 (8)
C18	0.1038 (18)	0.0631 (13)	0.0800 (16)	0.0407 (13)	0.0040 (13)	−0.0145 (11)
C19	0.0734 (14)	0.0559 (12)	0.0712 (14)	0.0292 (11)	0.0100 (11)	−0.0080 (10)
C20	0.0917 (18)	0.0732 (15)	0.0921 (19)	0.0143 (13)	0.0280 (15)	−0.0044 (14)
C21	0.132 (3)	0.100 (2)	0.0829 (19)	0.047 (2)	0.0413 (19)	0.0101 (16)
C22	0.108 (2)	0.099 (2)	0.0810 (19)	0.0477 (19)	−0.0079 (18)	−0.0167 (17)
C23	0.0765 (17)	0.0812 (18)	0.119 (3)	0.0129 (14)	−0.0030 (17)	−0.0133 (18)
C24	0.0812 (16)	0.0728 (15)	0.0953 (19)	0.0221 (13)	0.0228 (14)	0.0054 (13)
C25	0.0778 (13)	0.0437 (10)	0.0647 (12)	0.0206 (9)	0.0090 (10)	−0.0030 (9)
C26	0.0655 (12)	0.0383 (9)	0.0620 (11)	0.0132 (8)	0.0103 (9)	−0.0014 (8)
C27	0.0686 (13)	0.0488 (11)	0.0873 (15)	0.0067 (9)	0.0210 (11)	0.0144 (10)
C28	0.0607 (12)	0.0635 (13)	0.0987 (17)	0.0162 (10)	0.0291 (12)	0.0154 (12)
C29	0.0678 (13)	0.0503 (11)	0.0880 (15)	0.0219 (9)	0.0192 (11)	0.0110 (10)
C30	0.0898 (16)	0.0542 (12)	0.0903 (16)	0.0246 (11)	0.0363 (13)	0.0220 (11)
C31	0.0802 (14)	0.0577 (12)	0.0816 (15)	0.0263 (10)	0.0362 (12)	0.0118 (11)

### (2E)-1-[3,5-Bis(benzyloxy)phenyl]-3-(4-ethoxyphenyl)prop-2-en-1-one Geometric parameters (Å, º)

**Table:**

O1—C3	1.354 (3)	C14—C15	1.379 (3)
O1—C2	1.440 (3)	C15—C16	1.382 (2)
O2—C11	1.221 (2)	C15—H15	0.9300
O3—C16	1.368 (2)	C16—C17	1.384 (3)
O3—C18	1.419 (2)	C17—H17	0.9300
O4—C14	1.369 (2)	C18—C19	1.499 (3)
O4—C25	1.424 (2)	C18—H18A	0.9700
C1—C2	1.446 (4)	C18—H18B	0.9700
C1—H1A	0.9600	C19—C20	1.358 (4)
C1—H1B	0.9600	C19—C24	1.374 (4)
C1—H1C	0.9600	C20—C21	1.377 (4)
C2—H2A	0.9700	C20—H20	0.9300
C2—H2AB	0.9700	C21—C22	1.354 (5)
C3—C8	1.385 (3)	C21—H21	0.9300
C3—C4	1.388 (3)	C22—C23	1.364 (5)
C4—C5	1.365 (3)	C22—H22	0.9300
C4—H4	0.9300	C23—C24	1.383 (4)
C5—C6	1.392 (3)	C23—H23	0.9300
C5—H5	0.9300	C24—H24	0.9300
C6—C7	1.399 (3)	C25—C26	1.504 (2)
C6—C9	1.444 (3)	C25—H25A	0.9700
C7—C8	1.369 (3)	C25—H25B	0.9700
C7—H7	0.9300	C26—C31	1.376 (3)
C8—H8	0.9300	C26—C27	1.376 (3)
C9—C10	1.331 (3)	C27—C28	1.386 (3)
C9—H9	0.9300	C27—H27	0.9300
C10—C11	1.467 (3)	C28—C29	1.360 (3)
C10—H10	0.9300	C28—H28	0.9300
C11—C12	1.504 (3)	C29—C30	1.370 (3)
C12—C17	1.388 (3)	C29—H29	0.9300
C12—C13	1.399 (2)	C30—C31	1.382 (3)
C13—C14	1.388 (3)	C30—H30	0.9300
C13—H13	0.9300	C31—H31	0.9300
			
C3—O1—C2	117.52 (18)	O3—C16—C15	114.35 (17)
C16—O3—C18	117.54 (16)	O3—C16—C17	125.27 (16)
C14—O4—C25	119.51 (15)	C15—C16—C17	120.38 (18)
C2—C1—H1A	109.5	C16—C17—C12	119.58 (16)
C2—C1—H1B	109.5	C16—C17—H17	120.2
H1A—C1—H1B	109.5	C12—C17—H17	120.2
C2—C1—H1C	109.5	O3—C18—C19	107.89 (17)
H1A—C1—H1C	109.5	O3—C18—H18A	110.1
H1B—C1—H1C	109.5	C19—C18—H18A	110.1
O1—C2—C1	108.5 (2)	O3—C18—H18B	110.1
O1—C2—H2A	110.0	C19—C18—H18B	110.1
C1—C2—H2A	110.0	H18A—C18—H18B	108.4
O1—C2—H2AB	110.0	C20—C19—C24	118.8 (2)
C1—C2—H2AB	110.0	C20—C19—C18	120.3 (3)
H2A—C2—H2AB	108.4	C24—C19—C18	120.9 (3)
O1—C3—C8	117.02 (19)	C19—C20—C21	120.6 (3)
O1—C3—C4	123.8 (2)	C19—C20—H20	119.7
C8—C3—C4	119.2 (2)	C21—C20—H20	119.7
C5—C4—C3	119.6 (2)	C22—C21—C20	120.6 (3)
C5—C4—H4	120.2	C22—C21—H21	119.7
C3—C4—H4	120.2	C20—C21—H21	119.7
C4—C5—C6	122.75 (19)	C21—C22—C23	119.7 (3)
C4—C5—H5	118.6	C21—C22—H22	120.2
C6—C5—H5	118.6	C23—C22—H22	120.2
C5—C6—C7	116.49 (19)	C22—C23—C24	119.8 (3)
C5—C6—C9	122.29 (18)	C22—C23—H23	120.1
C7—C6—C9	121.22 (19)	C24—C23—H23	120.1
C8—C7—C6	121.5 (2)	C19—C24—C23	120.6 (3)
C8—C7—H7	119.2	C19—C24—H24	119.7
C6—C7—H7	119.2	C23—C24—H24	119.7
C7—C8—C3	120.5 (2)	O4—C25—C26	106.86 (15)
C7—C8—H8	119.8	O4—C25—H25A	110.4
C3—C8—H8	119.8	C26—C25—H25A	110.4
C10—C9—C6	127.10 (19)	O4—C25—H25B	110.4
C10—C9—H9	116.4	C26—C25—H25B	110.4
C6—C9—H9	116.4	H25A—C25—H25B	108.6
C9—C10—C11	123.09 (19)	C31—C26—C27	118.09 (18)
C9—C10—H10	118.5	C31—C26—C25	120.9 (2)
C11—C10—H10	118.5	C27—C26—C25	121.05 (19)
O2—C11—C10	121.00 (18)	C26—C27—C28	120.7 (2)
O2—C11—C12	119.63 (18)	C26—C27—H27	119.7
C10—C11—C12	119.36 (16)	C28—C27—H27	119.7
C17—C12—C13	120.41 (17)	C29—C28—C27	120.7 (2)
C17—C12—C11	117.54 (16)	C29—C28—H28	119.7
C13—C12—C11	122.00 (17)	C27—C28—H28	119.7
C14—C13—C12	118.88 (18)	C28—C29—C30	119.28 (19)
C14—C13—H13	120.6	C28—C29—H29	120.4
C12—C13—H13	120.6	C30—C29—H29	120.4
O4—C14—C15	114.51 (16)	C29—C30—C31	120.2 (2)
O4—C14—C13	124.79 (17)	C29—C30—H30	119.9
C15—C14—C13	120.70 (16)	C31—C30—H30	119.9
C16—C15—C14	120.01 (18)	C26—C31—C30	121.0 (2)
C16—C15—H15	120.0	C26—C31—H31	119.5
C14—C15—H15	120.0	C30—C31—H31	119.5
			
C3—O1—C2—C1	177.2 (3)	C18—O3—C16—C15	−177.5 (2)
C2—O1—C3—C8	179.8 (2)	C18—O3—C16—C17	2.7 (3)
C2—O1—C3—C4	−0.1 (4)	C14—C15—C16—O3	−177.57 (19)
O1—C3—C4—C5	−179.4 (2)	C14—C15—C16—C17	2.2 (3)
C8—C3—C4—C5	0.7 (3)	O3—C16—C17—C12	178.65 (19)
C3—C4—C5—C6	0.0 (3)	C15—C16—C17—C12	−1.1 (3)
C4—C5—C6—C7	−0.9 (3)	C13—C12—C17—C16	−0.5 (3)
C4—C5—C6—C9	179.5 (2)	C11—C12—C17—C16	177.29 (18)
C5—C6—C7—C8	1.1 (3)	C16—O3—C18—C19	179.5 (2)
C9—C6—C7—C8	−179.4 (2)	O3—C18—C19—C20	−78.4 (3)
C6—C7—C8—C3	−0.4 (4)	O3—C18—C19—C24	103.6 (3)
O1—C3—C8—C7	179.6 (2)	C24—C19—C20—C21	0.4 (3)
C4—C3—C8—C7	−0.5 (4)	C18—C19—C20—C21	−177.6 (2)
C5—C6—C9—C10	−5.9 (3)	C19—C20—C21—C22	0.2 (4)
C7—C6—C9—C10	174.6 (2)	C20—C21—C22—C23	−0.5 (4)
C6—C9—C10—C11	179.11 (19)	C21—C22—C23—C24	0.2 (4)
C9—C10—C11—O2	−11.5 (3)	C20—C19—C24—C23	−0.7 (3)
C9—C10—C11—C12	169.80 (19)	C18—C19—C24—C23	177.3 (2)
O2—C11—C12—C17	−9.3 (3)	C22—C23—C24—C19	0.4 (4)
C10—C11—C12—C17	169.33 (19)	C14—O4—C25—C26	176.21 (18)
O2—C11—C12—C13	168.4 (2)	O4—C25—C26—C31	70.9 (2)
C10—C11—C12—C13	−13.0 (3)	O4—C25—C26—C27	−109.3 (2)
C17—C12—C13—C14	1.0 (3)	C31—C26—C27—C28	−0.6 (3)
C11—C12—C13—C14	−176.69 (18)	C25—C26—C27—C28	179.60 (19)
C25—O4—C14—C15	−174.2 (2)	C26—C27—C28—C29	−0.6 (3)
C25—O4—C14—C13	5.3 (3)	C27—C28—C29—C30	1.0 (4)
C12—C13—C14—O4	−179.38 (19)	C28—C29—C30—C31	−0.3 (4)
C12—C13—C14—C15	0.1 (3)	C27—C26—C31—C30	1.3 (3)
O4—C14—C15—C16	177.83 (19)	C25—C26—C31—C30	−178.9 (2)
C13—C14—C15—C16	−1.7 (3)	C29—C30—C31—C26	−0.9 (4)

### (2E)-1-[3,5-Bis(benzyloxy)phenyl]-3-(4-ethoxyphenyl)prop-2-en-1-one Hydrogen-bond geometry (Å, º)

*Cg*1 is the centroid of the C26–C31 ring.

**Table:**

*D*—H···*A*	*D*—H	H···*A*	*D*···*A*	*D*—H···*A*
C1—H1*A*···O4^i^	0.96	2.64	3.227 (3)	119
C25—H25*B*···*Cg*1^ii^	0.97	2.68	3.398 (2)	132

Symmetry codes: (i) *x*−1, *y*, *z*−1; (ii) −*x*+1, −*y*, −*z*+1.

### IR spectroscopic data (cm^–1^)

**Table:**

Assignments	1
δ (CH_2_) rocking	696(m)
δ (C-Cl)	-
δ (C-H)(Ar)	835(m)
ν (C-O-C)	1049(s)
δ (C-H) of –OCH_2_CH_3_	1328(m)
δ (C-H) of –OCH_3_	-
ν (C-C)	1433(m)
ν (C=C)	1568(s)
ν (C=O)	1651(s)
ν (C-H) (aliphatic)	2934(w)
ν (C-H)(aromatic)	3032(w)

ν -stretching, δ-bending, a-antisymmetry, s-symmetry, (w)-weak intensity, (m)-medium peak, (s)-strong peak

## References

[bb1] Ashfaq, M., Tahir, M. N., Muhammad, S., Munawar, K. S., Ali, A., Bogdanov, G. & Alarfaji, S. S. (2021). *ACS Omega*, **6**, 31211–31225. 10.1021/acsomega.1c04884 PMC8613867 34841164

[bb2] Bruker (2017). *APEX2*, and *SAINT*. Bruker AXS Inc., Madison, Wisconsin, U. S. A.

[bb3] Farrugia, L. J. (2012). *J. Appl. Cryst.* **45**, 849–854.

[bb4] Liu, W., He, M., Li, Y., Peng, Z. & Wang, G. (2022). *J. Enzyme Inhib. Med. Chem.* **37**, 9–38. 10.1080/14756366.2021.1976772 PMC8667932 34894980

[bb6] Mai, C. W., Yaeghoobi, M., Abd-Rahman, N., Kang, Y. B. & Pichika, M. R. (2014). *Eur. J. Med. Chem.* **77**, 378–387. 10.1016/j.ejmech.2014.03.002 24675137

[bb7] Sheldrick, G. M. (2015*a*). *Acta Cryst.* A**71**, 3–8.

[bb8] Sheldrick, G. M. (2015*b*). *Acta Cryst.* C**71**, 3–8.

[bb10] Spackman, P. R., Turner, M. J., McKinnon, J. J., Wolff, S. K., Grimwood, D. J., Jayatilaka, D. & Spackman, M. A. (2021). *J. Appl. Cryst.* **54**, 1006–1011. 10.1107/S1600576721002910 PMC8202033 34188619

[bb11] Spek, A. L. (2020). *Acta Cryst.* E**76**, 1–11. 10.1107/S2056989019016244 PMC6944088 31921444

[bb12] Zhuang, C., Zhang, W., Sheng, C., Zhang, W., Xing, C. & Miao, Z. (2017). *Chem. Rev.* **117**, 7762–7810. 10.1021/acs.chemrev.7b00020 PMC6131713 28488435

